# Real Time Live Imaging of Phytopathogenic Bacteria *Xanthomonas campestris* pv. *campestris* MAFF106712 in ‘Plant Sweet Home’

**DOI:** 10.1371/journal.pone.0094386

**Published:** 2014-04-15

**Authors:** Chiharu Akimoto-Tomiyama, Ayako Furutani, Hirokazu Ochiai

**Affiliations:** 1 Plant-Microbe Interaction Research Unit, Division of Plant Sciences, National Institute of Agrobiological Sciences, Tsukuba, Japan; 2 Gene Research Center, Ibaraki University, Inashiki, Japan; University of Wisconsin-Milwaukee, United States of America

## Abstract

Xanthomonas is one of the most widespread phytobacteria, causing diseases on a variety of agricultural plants. To develop novel control techniques, knowledge of bacterial behavior inside plant cells is essential. *Xanthomonas campestris* pv. *campestris*, a vascular pathogen, is the causal agent of black rot on leaves of Brassicaceae, including *Arabidopsis thaliana*. Among the *X. campestris* pv. *campestris* stocks in the MAFF collection, we selected *Xcc*MAFF106712 as a model compatible pathogen for the *A. thaliana* reference ecotype Columbia (Col-0). Using modified green fluorescent protein (AcGFP) as a reporter, we observed real time *Xcc*MAFF106712 colonization *in planta* with confocal microscopy. AcGFP-expressing bacteria colonized the inside of epidermal cells and the apoplast, as well as the xylem vessels of the vasculature. In the case of the type III mutant, bacteria colonization was never detected in the xylem vessel or apoplast, though they freely enter the xylem vessel through the wound. After 9 days post inoculation with *Xcc*MAFF106712, the xylem vessel became filled with bacterial aggregates. This suggests that *Xcc* colonization can be divided into main four steps, (1) movement in the xylem vessel, (2) movement to the next cell, (3) adhesion to the host plant cells, and (4) formation of bacterial aggregates. The type III mutant abolished at least steps (1) and (2). Better understanding of *Xcc* colonization is essential for development of novel control techniques for black rot.

## Introduction

When phytobacteria in the environment find a plant host, they dock and bind to host surface receptors [1], multiply on the plant surface, and then attempt to invade the endophytic space through stomata, hydathodes or wounds. Phytopathogens may produce a variety of virulence factors such as cell wall-degrading enzymes, extracellular- and lipo-polysaccharides, and a type III secretion (TTS) system with associated type III effector proteins (T3Es) that contribute to the ability of the bacterium to parasitize the host [2], [3]. Extracellular enzymes capable of degrading plant cell components may be required to overcome plant defense responses, to allow bacteria to move into uncolonized plant tissues, and to mobilize plant polymers for nutritional purposes [4]–[6]. Bacterial extracellular polysaccharides (EPS) such as xanthan, alginate, and amylovoran induce plant susceptibility to the pathogens by suppressing basal defenses such as callose deposition [7], participating in biofilm formation [8], and potentially protecting bacteria from the stresses of desiccation and other host defenses [9]. The TTS system is a protein secretion apparatus used by animal and plant pathogens to deliver T3E virulence proteins directly into host cells, where they can modulate the host’s physiology and manipulate the host immune system [10]–[12]. Coincident with T3E delivery, if the effectors are not recognized by resistance proteins in the host, the pathogens multiply quickly and eventually cause disease. Phytobacteria are classified into two major groups - mesophyllic and vascular pathogens - based on their primary growth location. Mesophyllic pathogens such as *Pseudomonas* [13] and *Xanthomonas campestris* pv. *vesicatoria* [14] grow mainly in the appoplast. Vascular pathogens like *Ralstonia* [15] and *X*. *campestris* pv. *campestris* [14], *X. oryzae* pv. *oryzae* [14] grow mainly in the vascular system. Detailed observations of various type of invading pathogens in their host plant have been published [16]–[21]. Han et al developed efficient methods for visualizing fluorescent *Xanthomonas oryzae* pv. *oryzae* in rice [22]. However, there is little knowledge of vascular pathogen biology in a dicot plant. *Xanthomonas campestris* pv. *campestris* (*Xcc*) is a vascular, seed-borne pathogen distributed worldwide and the causal agent of black rot on the leaves of economically-important Brassicaceae such as cabbage, mustard, and radish [14]. *Xcc*, along with the model Brassicaceae *A. thaliana*, make a good system to study a vascular pathogen interaction because of the abundant genetic resources. To our knowledge, there is no report of real-time imaging of *Xcc* bacteria inside a plant host. To develop novel anti-phytobacterial agents and control techniques, behavioral analysis of *Xcc* in its host is critical.

Currently, there are four complete *X. campestris* pv. *campestris* genomes available [23]–[26]. While *Xcc*ATCC33913 is compatible in the ecotype San Feliu-2 (Sf-2) [27], all four sequenced strains are incompatible with the reference ecotype Columbia (Col-0) [28]. It is hypothesized that two type III effectors, XopAC and XopAM, work as avirulence factors in Col-0 [28]. In order to investigate the compatible interaction of *Xcc* and Arabidopsis, we searched for an *Xcc* which is virulent on Col-0. Among the 15 MAFF *Xcc* collection isolates stocked at National Institute of Agrobiological Sciences in Japan, we found that *Xcc*MAFF106712 caused disease on Col-0. Using AcGFP-tagged *Xcc*MAFF106712, we observed real-time bacterial behavior with confocal microscopy. The bacteria freely moved through the xylem vessels, and proliferated in the xylem vessels and multiplied apoplast and inside epidermal cells of Col-0. In contrast, in the incompatible interaction in ecotype Sf-2, *Xcc*MAFF106712 failed to spread and we observed a robust HR-like defense response. Moreover, in a TTS system mutant of *Xcc*MAFF106712 inoculated on Col-0, movement of the bacteria and colonization in the xylem vessel were strictly inhibited, indicating type III effectors have a role during invasion of host cells. The temporal dynamics of infection contribute to an understanding that can help control the phytobacteria *Xcc*.

## Materials and Methods

### Bacterial Strains

**Figure 1:**
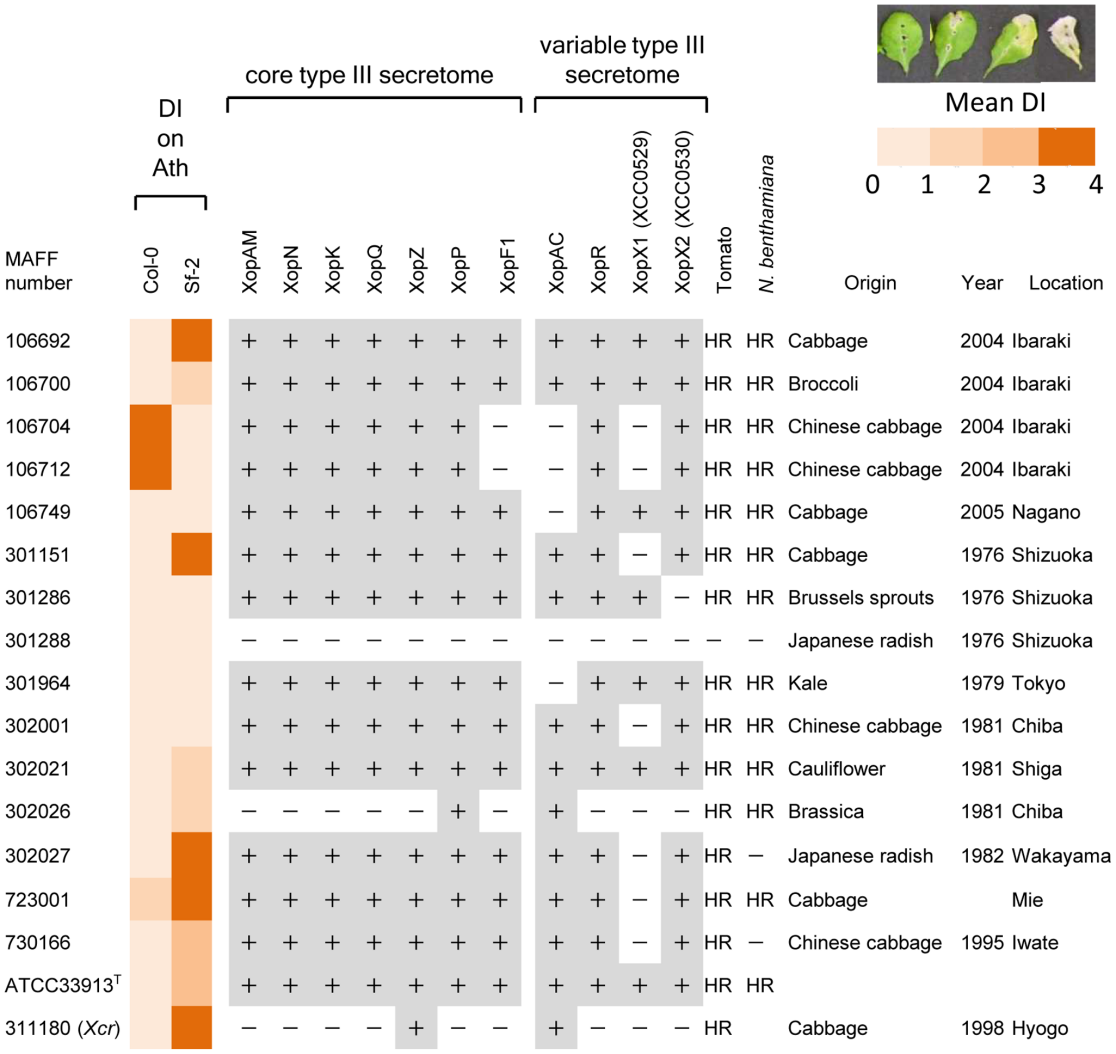
Interactions between two *Arabidopsis* ecotypes vs. 16 *X. campestris* pv. *campestris* isolates. All strains are shown by their MAFF collection number except for ATCC33913 type strain (ATCC33913^T^), and the distribution of genes coding for type III secreted proteins is indicated. The pathogenicity (mean disease index at 7 dpi) of these *X. campestris* pv. *campestris* strains on Arabidopsis ecotypes Col-0 and Sf-2 is indicated by color coding (0 to 1 indicates no symptoms, 1 to 2 indicates weak chlorosis, 2 to 3 indicates strong chlorosis, and 3 to 4 indicates necrosis as shown in the upper panel). Bacteria were inoculated by piercing the central leaf vein three times with a D200 pipette tip that had been dipped in a bacterial suspension (10^9 ^cfu mL^−1^). The presence or absence of a homologous T3SP gene sequence was determined by PCR. Strains were also inoculated on nonhost tomato (momotaro) and *N. benthamiana*. HR, hypersensitive response; -, no HR. Dark-gray squares and+represent the presence of the corresponding genes with both primer combinations at the expected sizes, whereas white squares and - represent the absence of PCR amplification or PCR with a different amplicon size.

*X. campestris* pv. *campestris* strains used in this study are listed in Figure 1. All MAFF *Xcc* collection isolates are stocked at National Institute of Agrobiological Sciences Genebank in Japan. *X. campestris* pv. *campestris* cells were grown at 28°C in nutrient broth-yeast extract medium [27]. *Escherichia coli* cells were grown on Luria-Bertani medium at 37°C. For solid media, agar was added at a final concentration of 1.5% (wt vol^−1^). Antibiotics were used at the following concentrations: for *X*. *campestris* pv. *campestris*, 50 μg ml^−1^ rifampin, 50 μg ml^−1^ kanamycin, and 40 μg ml^−1^ spectinomycin, and for *E. coli*, 50 μg ml^−1^ ampicillin, 25 μg ml^−1^ kanamycin, 40 μg ml^−1^ spectinomycin.

### Construction of a *hrcC*-deletion Mutant of *X. campestris* pv. *campestris*

Marker exchange mutagenesis of *X. campestris* pv. *campestris* strain MAFF106712 was conducted using the plasmid pUCdeltahrcCKm [12]. The resulting strain was named 106712ΔHrcC. Marker exchange mutagenesis was confirmed by genomic Southern blot analysis (data not shown).

### Construction of AcGFP-expressing *X. campestris* pv. *campestris*

The AcGFP gene was amplified from pAcGFP1 (Takara, Tokyo, Japan) using a primer set with appropriate restriction site: AcGFP_Fp; 5′-aaaaagcttcatggtgagcaagggcgccgagctgttcacc-3′ and AcGFP_R; 5′-aagaattctcacttgtacagctcatccatgccg-3′. After treatment with *Eco*RI and *Hin*dIII, the fragment was inserted to *Eco*RI/*Hin*dIII-digested pUC18 [29] to give pUC18AcGFP. Then, an approximately 0.7-kb *Eco*RI/*Hin*dIII fragment containing the AcGFP gene was inserted to a broad-host-range vector pHM1 [30] to give pHMAcGFP which expresses the AcGFP gene under the control of the *lacZ* promoter. Plasmid pHMAcGFP was introduced into *X. campestris* pv. *campestris*, and the transformants were used as AcGFP-expressing strains.

### Plant Material, Growth Conditions, and Infection Tests

*Arabidopsis* plants were grown on soil in pots as described previously [12]. Natural variation in *Xcc* pathogenicity was assayed on the *A. thaliana* natural accessions Col-0 and Sf-2 by piercing inoculation of a bacterial suspension at 10^9^ CFU ml^−1^ as described previously [12], [27] with some modifications. Briefly, 1 μl of the bacteria suspension was put on the central vein in three spots per leaf, by making a tiny wound with a D200 pipette tip (GILSON, WI, USA). Each of the 17 strains was tested on 4 plants per ecotype (Col-0 and Sf-2) and 4 leaves per plant. Three independent repetitions were done in 16 blocks. After inoculation, plants were covered by a plastic film and kept at nearly 100% relative humidity for 24 hours. Disease development was scored at 7 dpi using a disease index ranging from 0 (no symptoms) to 4 (full leaf necrosis) as described previously [27]. Nonhost tomato and *Nicotiana benthamiana* plants were grown and inoculated at an optical density of 0.4 at 600 nm (OD600) as previously described [31]. The HR was scored 36 h post infiltration.

### PCR-based Detection of T3E Genes in *X*. *campestris* pv. *campestris*

Genomic DNA (gDNA) extractions were prepared for each strain as described by the manufacturer [32], and used for PCR analyses. The presence of T3E genes was determined using 2 pairs of gene-specific primers designed for the *X*. *campestris* pv. *campestris* ATCC33913-orthologs. For each gene, one of the primer pairs amplified the full-length T3SP DNA sequence, while the other one amplified a shorter sequence of ca. 300 bp usually in the 5′ coding region. All oligonucleotide sequences are listed in Table S1. A reaction was considered positive (the gene was present) if a single clear band with the expected size was observed after separation on 1% agarose gel.

### Determination of in Planta Bacterial Populations

Twenty-four leaves from different 8 plants were inoculated by piercing the leaves with an *X*. *campestris* pv. *campestris* suspension of 10^9^ CFU ml^−1^. Three inoculated leaves were sampled in triplicate at 0, 3, and 7 days after inoculation. Fresh tissues were homogenized in 500 μl sterile water. Serial dilutions of the homogenates were performed, and a 10 μl drop was spotted for each dilution on plates supplemented with appropriate antibiotics. The plates were incubated at 28°C for 48 h, and colonies were counted for spots containing 1 to 30 colonies. Experiments were performed at least three times.

### Microscopy

A confocal laser-scanning microscope (CLSM) TCS SP5 (Leica Microsystems, Solms, Germany) was used to visualize the AcGFP-expressing bacteria. Bacteria were inoculated on Arabidopsis leaves by piercing three holes in a central vein, and at the indicated times the leaf was detached from the plant and mounted on the glass slide with deionized water and a coverslip. Dual-colour images were acquired by sequentially scanning with settings optimal for AcGFP (488 nm excitation (Ex), 505 nm long pass emission (Em)), and chlorophyll autofluorescence (514 nm Ex and 650–700 nm Em). The detached *Arabidopsis* leaf was dipped in 0.1 μg/μl membrane-selective dye N-(3-Triethylammoniumpropyl)-4-(6-(4-(Diethylamino)phenyl)hexatrienyl)Pyridinium Dibromide (FM4-64) (Invitrogen) in deionized water for 30 minutes at room temperature. After brief wash in deionized water, FM4-64 (488 nm Ex, 650–700 nm Em) and AcGFP-expressing bacteria (488 nm Ex, 505 nm long pass Em) were detected using a CLSM. Cross talk between the channels in this setup was always monitored and, in all cases, was negligible. We also acquired a bright field image using a photo-multiplier detector measuring the transmitted light. For time-lapse microscopy, the CLSM was programmed to acquire an optical section (at the same plane) every 1 s. Microscope power settings were adjusted to optimize contrast for each individual image. Images were collected using LCS software (Leica) and imported into Photoshop (Adobe) for brightness and contrast adjustments and assembly of the composite figure for publication. All of the photograph in the figure were picked from one optical section. All Movies in supporting information are shown as their original movies collected by the LCS software.

### Staining of Dead Cells with Trypan Blue

Staining with trypan blue was performed, with slight modifications, as described previously [33]. Briefly, detached leaves were soaked in trypan blue [75% ethanol, 8.3% TE-saturated phenol (pH 8.0), 8.3% glycerol, 8.3% lactic acid and 0.17 g ml^−1^ trypan blue]. The leaves were then boiled for 1 min and left overnight at room temperature. Finally, the leaves were washed several times with 2.5 g ml^−1^ chloral hydrate and observed under stereomicroscope (SMZ745T, Nikon, Tokyo, Japan) or microscope (ECLIPSE E100, Nikon) equipped with a camera (ICU-200, Microscope Network, Kawaguchi, Japan). Images were collected using ICU-200 software (Microscope Network) and imported into Photoshop (Adobe) for brightness and contrast adjustments and assembly of the composite Figure for publication.

## Results

### Screening of Col-0 Compatible *X. campestris* pv. *campestris* Strains

**Figure 2:**
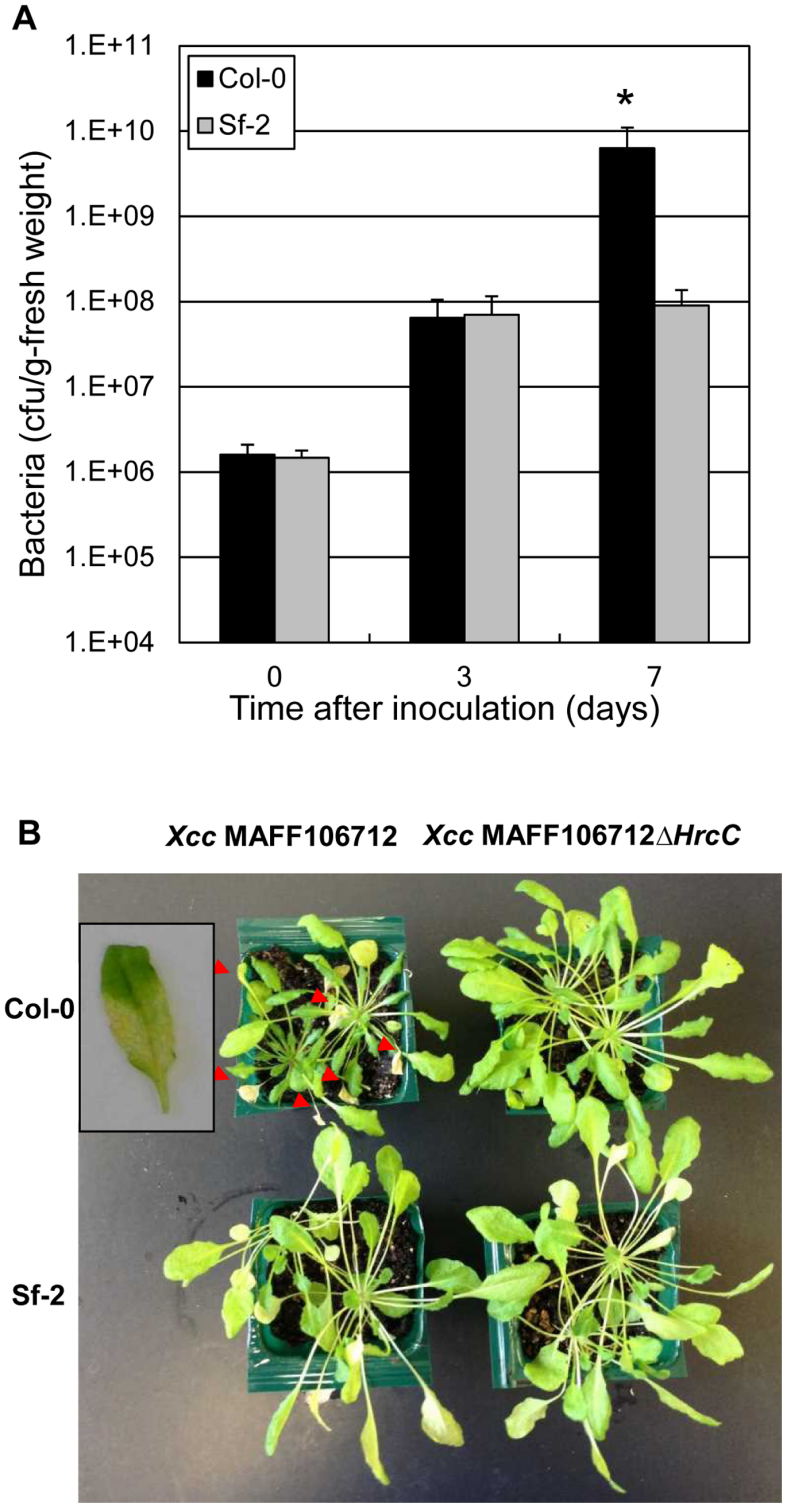
*Xcc*MAFF106712 is virulent for Arabidopsis ecotypes Col-0 plants in a type III dependent manner. (A) In planta bacterial growth of *Xcc*MAFF106712 after inoculation on Arabidopsis ecotypes Col-0 (solid) and Sf-2 (gray). Twenty-four leaves from 8 different plants were inoculated by piercing the leaves with an *Xcc*MAFF106712 suspension of 10^9^ cfu mL^−1^. Enhanced bacterial multiplication in Col-0 compared to Sf-2 was significant (*P<0.001, Student’s t test). Results are representative of at least three experiments per condition. (B) *Xcc* MAFF106712 pathogenicity on Col-0 is dependent on the type III secretion system. Bacteria (*Xcc*MAFF106712 and MAFF106712Δ*hrcC*) were inoculated on Col-0 and Sf-2 plants and photographs were taken 15 days post inoculation. Black rot phenotype of Col-0 plant 7 days post *Xcc*MAFF106712 inoculation is shown in the window. At 7 dpi, some infected leaves are still green and some of the leaves start showing the disease phenotype. We used mixed up them for the growth assay shown in the panel A. Red arrows indicate necrotic leaves caused by bacteria inoculation.

Natural variation of *X. campestris* pv. *campestris* pathogenicity was assayed on the *A. thaliana* ecotypes Col-0 and Sf-2 by piercing inoculation, in which disease development was observed 7 days post inoculation (Figure 1). Two *Xcc* strains, MAFF106704 and MAFF106712, caused strong leaf necrosis on Col-0. In contrast, these two strains did not show any visible symptoms on Sf-2. Since these two strains have the same phenotype and were originally isolated from the same location, we suspect they are closely related. To quantify bacterial growth inside the plant, *Xcc*MAFF106712 was inoculated by piercing inoculation [27] on both Col-0 and Sf-2 (Figure 2A). Three days post inoculation, *Xcc* MAFF106712 bacteria had proliferated to 100 times the initial concentration in both Col-0 and Sf-2. Seven days post inoculation, however, the growth in Col-0 had significantly overtaken that in Sf-2 plants and some of the inoculated leaves showed black rot phenotype as shown in Figure 2B. After 15 days, as shown in Figure 2B, inoculated leaves of the Col-0 showed lethal black rot symptoms, while the Sf-2 leaves showed no symptoms. Additionally, the rosette growth of the inoculated Col-0 was stunted relative to Sf-2 (Figure 2B).

To test if the virulent interaction was type III dependent, we made the *hrcC* deletion mutant and inoculated Col-0. As shown in Figure 2B, the necrosis of infected leaves and the strong growth defect caused by the wild type strain were absent for type III deficient mutants. As shown in Figure 2B, both *hrcC* and wild type versions of the *Xcc*MAFF106712 were incompatible with Sf-2, showing no visible phenotype at 15 days post inoculation. From these results, we conclude that *Xcc*MAFF106712 is virulent on Col-0 plants in a type III dependent manner.

### *X. campestris* pv. *campestris* Strains Harbor a Variable Predicted type III Secretome

To elucidate whether one or more type III effectors is responsible for the virulence, existence of core type III effectors (XopAM, XopN, XopK, XopQ, XopZ and XopF1) and variable type III effectors (XopAC, XopR, XopX1 and XopX2) was confirmed by PCR amplification. As shown in Figure 1, XopF1, XopAC and XopX1 were missing in both *Xcc* MAFF106704 and MAFF106712 strains. This result supports our conclusion that these strains are closely related. The 16 strains, including *Xcc*ATCC33913 type strains, were inoculated on the non-hosts tomato and *N. benthamiana*, and development of HR was observed. As shown in Figure 1, most of the strains showed HR. *Xcc*MAFF301288 failed to cause HR on either tomato or *N. benthamiana*, while *Xcc*MAFF302027 and MAFF730166 did not cause HR on *N. benthamiana*.

### Real Time Imaging of *X. campestris* pv. *campestris* MAFF106712 Inside a Compatible Plant Host

**Figure 3:**
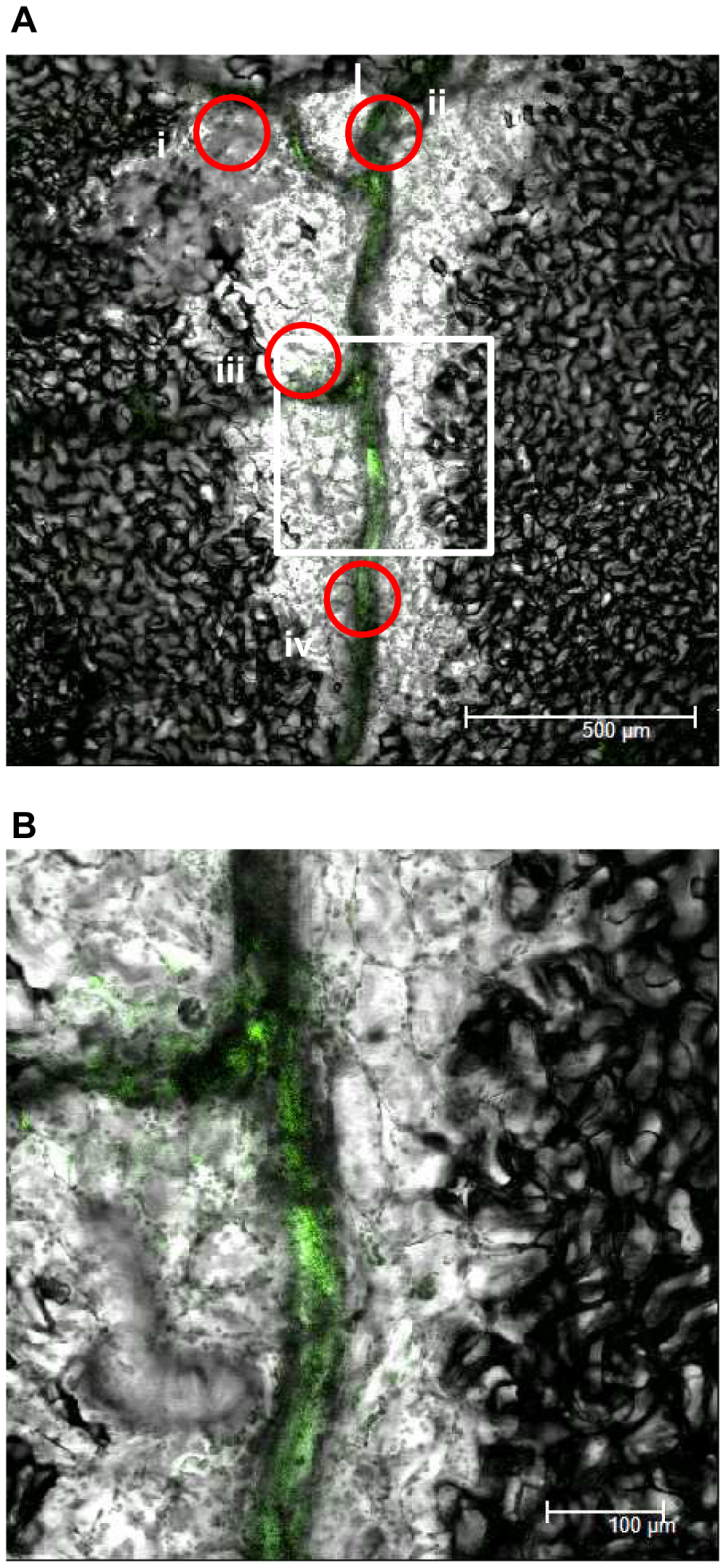
An AcGFP-expressing *Xcc*MAFF106712 bacteria colonized distal to the inoculated site at 6 days post inoculation. An inoculated leaf was detached from the plant and observed by a CLSM. Shown is the merged image of green fluorescence (green) and bright field. (A) Image shown around the inoculation site (I) in a low magnification. The red circles indicates the approximate location of the images taken in this manuscript. i, around (I); ii, central vein around (I); iii, distal to (I); iv, central vein distal to (I). White square showed the image shown in B Bar, 500 μm. (B) Image shown at the white square part of panel A in a higher magnification. Bar, 100 μm.

**Figure 4:**
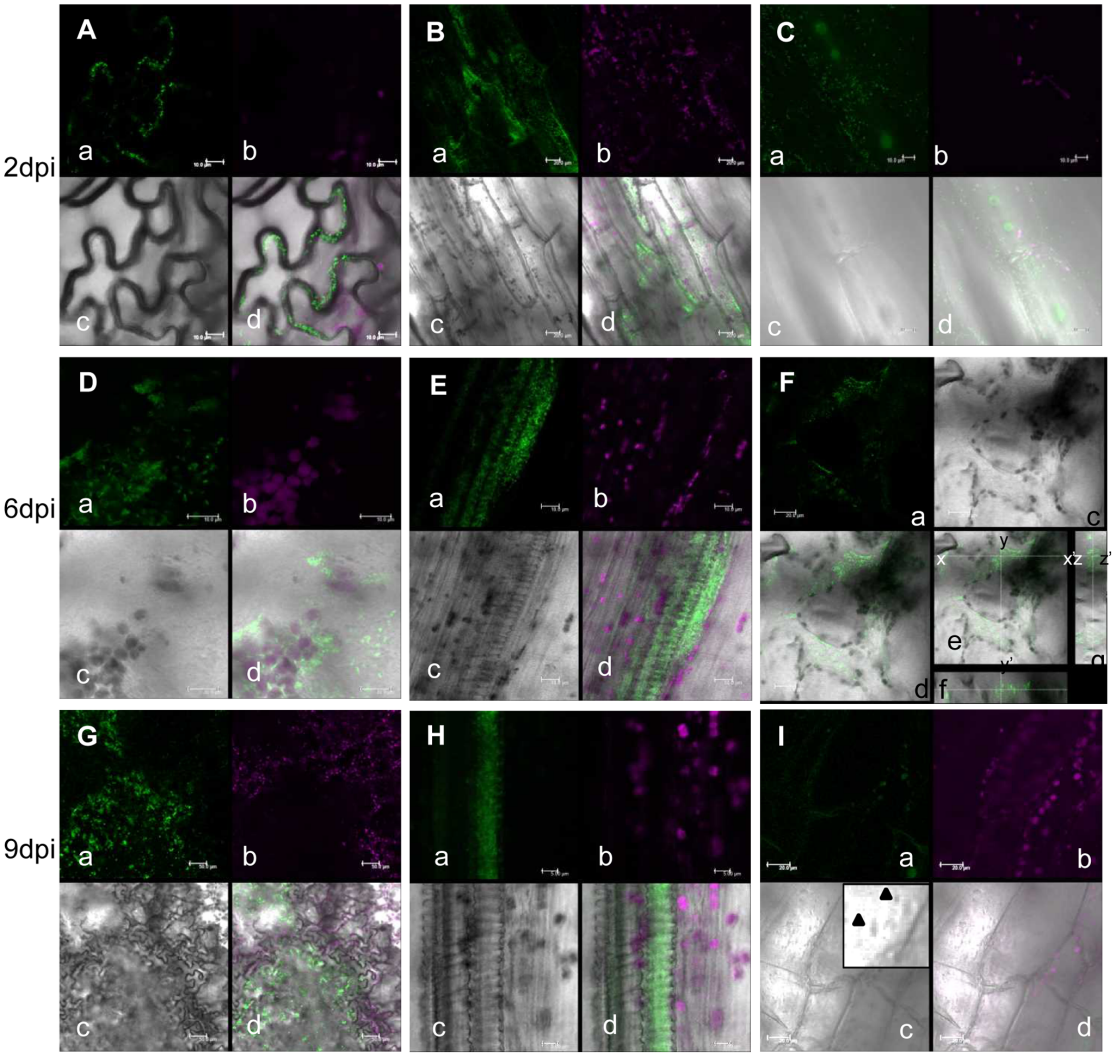
Time course extension of AcGFP-expressing *Xcc*MAFF106712 in Col-0 plant. Localization of AcGFP-expressing XccMAFF106712 (A to H) and *Xcc*MAFF106712 (I) in an Arabidopsis Col-0 plant. Leaf surface (A, D and G), central vein (B, C and I), the xylem vessel of the central vein (E, H) and mesophyll cells (F) were observed by confocal microscopy. Photographs were taken at 2 days (A, B and C), 6 days (D, E and F) and 9 days (G, H and I) after inoculation. Location indicated in Figure 2 : i,(A, I); ii, (B, C); iii, (D, F and G); iv, (E, H). Green fluorescent bacteria spread both in the cytosol of leaf epidermal cells, apoplast and the xylem vessel of the vein. a, green fluorescence (green); b, chlorophyll autofluorescence (red); c, bright field; d, merged image; e, same as d in panel F showing cross sectional line; f, cross-sectional view of c along the line x-x′; g, cross-sectional view of c along the line y-y′. X-x′, 158 μm; y-y′, 158 μm; z-z′, 32.8 μm. Bars, 10 μm (ACDE); 20 μm (BFI); 50 μm(G); 5 μm(H). Whole z series photos for panel F available at supplemental movie S5.

**Figure 5:**
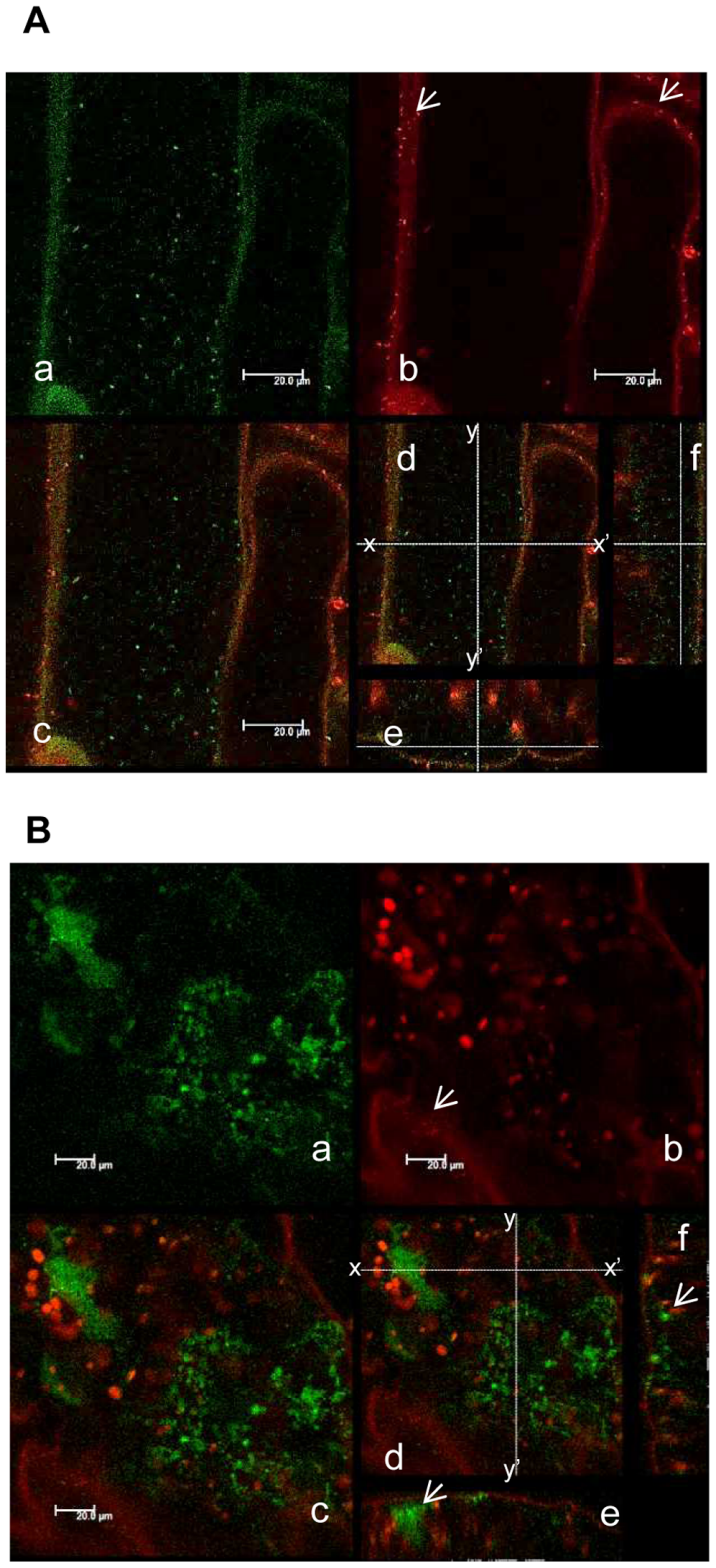
AcGFP-expressing *Xcc*MAFF106712 colonized in both leaf epidermal cells and the intercellular space in a Col-0 plant. An inoculated leaf was detached at the indicated time point and plasma membrane was stained with FM4-64. a, green fluorescence (green); b, FM4-64 and chlorophyll auto fluorescence are shown in red; c, merged image; d, same as panel c showing cross sectional line for e and f; e, cross-sectional view of c along the line x-x′ on d; f, cross-sectional view of c along the line y-y′ on d. (A) Epidermal cells of the central vein approximately 100 μm away from the initial inoculation edge in 3 dpi plant. The white arrow in panel b shows bacteria in plant surface that were stained with FM4-64. The bottom of the panel e and right side of the panel f shows plant surface. X-x′, 117 μm; y-y′, 117 μm; z-z′, 44 μm. Bars, 20 μm. (B) Epidermal cells approximately 600 μm away from the initial inoculation edge in the 9 dpi plant. The white arrow in panel b shows the stained bacteria on the surface of the epidermal cells. The white arrow in panel e shows aggregated cells in the intercellular space. The white arrow in panel f shows bacteria colonizing the epidermal cells. The upper side of the panel e and left side of the panel f shows the plant surface. X-x′, 180 μm; y-y′, 180 μm; z-z′, 20.5 μm. Bars, 20 μm.

**Figure 6:**
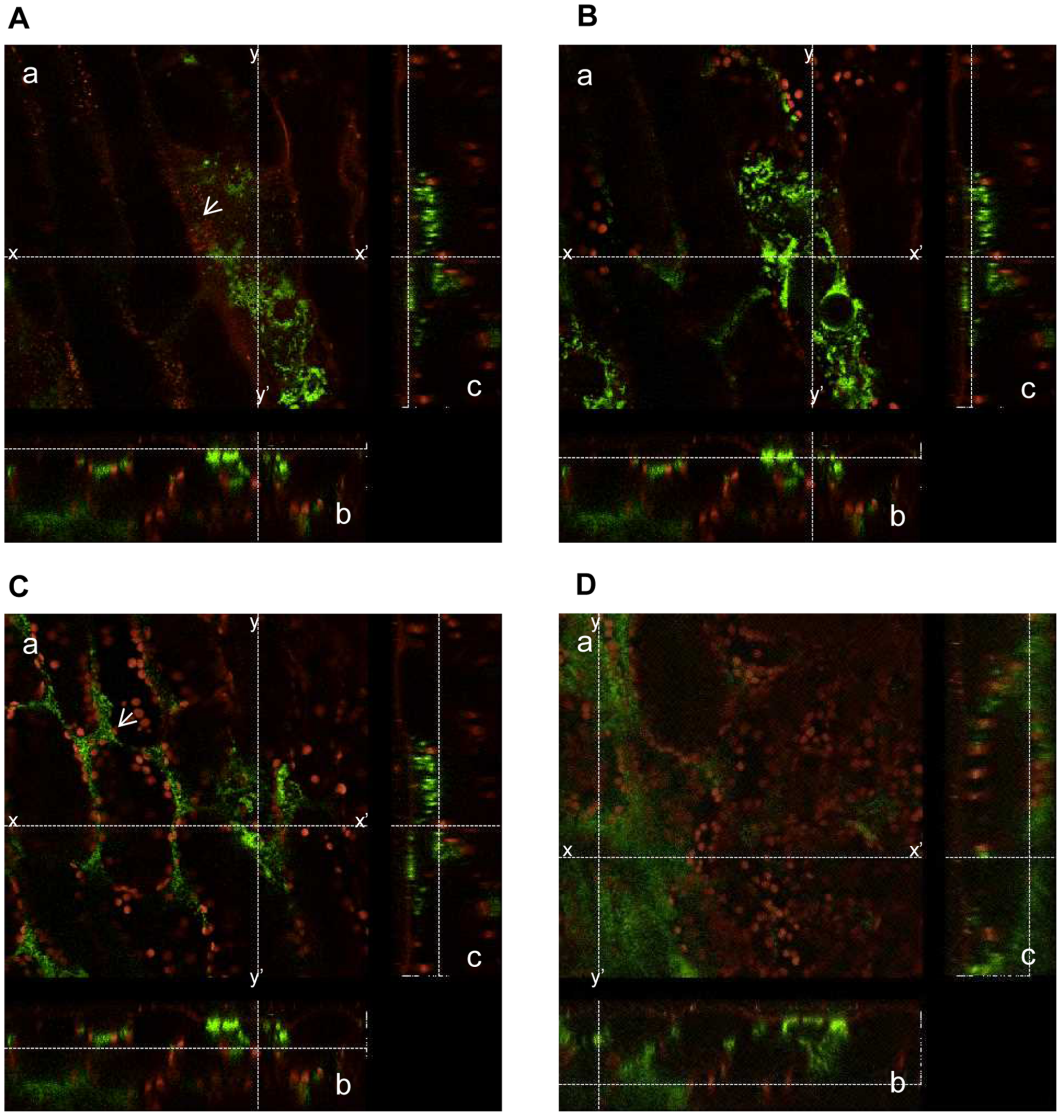
AcGFP-expressing *Xcc*MAFF106712 colonized the bundle sheath cells in a Col-0 plant. An inoculated leaf was detached at 9 days post inoculation and the plasma membrane was stained with FM4-64. Images shown are from the central vein approximately 3 mm away from the initial inoculation edge. a, merged image of the green fluorescence (green) and FM4-64 and chlorophyll auto fluorescence (red); b, cross-sectional view of a along the line x-x′ on a; c, cross-sectional view of a along the line y-y′ on a. X-x′, 240 μm; y-y′, 240 μm; z-z′, 80 μm. Upper side of the panel e and left side of the panel f showing the plant surface. A z-stack CLMS image is shown in supplemental Movie S7. (A) Cell surface. The white arrow in panel a shows bacteria at the surface that were stained with FM4-64. (B) Bacteria colonizing the epidermal cell. (C) Bacteria colonizing the apoplast. (D) Bacteria colonizing bundle sheath cells.

In order to observe *Xcc* behavior inside a compatible plant host, bacteria expressing commercially available engineered green fluorescent protein (AcGFP, Takara Bio, Tokyo, Japan) which increases the translational efficiency were developed and inoculated on Arabidopsis plants. The AcGFP-tagged *Xcc*MAFF106712 retained compatibility with Col-0, and caused the same plant growth defect 15 days post inoculation (data not shown). Strong positive correlation between fluorescence intensity and bacterial density (OD) was observed (data not shown), indicating each bacterium had a similar fluorescence intensity. In addition, colony forming units on plates both with and without antibiotics to select for the presence of the AcGFP-harboring plasmid were similar (data not shown), indicating that loss of the AcGFP plasmid is rare. The AcGFP-expressing *Xcc*MAFF106712 was inoculated on Col-0, and then AcGFP fluorescence was observed by CLSM. Six days after inoculation, AcGFP-expressing bacteria had multiplied distal to the inoculation site (Figure 3). A time course of detailed observation in epidermal cells, apoplast and xylem vessels is shown in Figure 4. Two days after inoculation, AcGFP-expressing bacteria had multiplied both outside and inside the epidermal cells around the inoculation site (Figures 4A, B, Movie S2) and inside surface cells on veins distal to the inoculation site (Figure 4C, Movie S1). At 6 days, the bacteria had multiplied inside both epidermal cells (Figures 3, 4D) and the xylem vessel (Figures 3, 4E, Movies S3, S4) and apoplast (Figures 3, 4F, Movie S5). The AcGFP-expressing bacteria multiplied inside epidermal cells (Figure 4G), and also multiple sites in the xylem vessel were filled with bacteria (Figure 4H) at 9 days after inoculation. Those results suggest that the xylem vessel was not only a main colonization place of *Xcc* but also a main gateway. No fluorescence was observed when untagged wild-type bacteria were inoculated, although single bacteria-sized objects were observed under bright field (Figure 4I), indicating the fluorescence signal we describe originated from AcGFP-tagged bacteria. To visualize the plant cell shape, the plasma membrane was stained by FM4-64. As shown in Figure 5, plant plasma membrane and external exogenous bacteria were stained, while endogenous bacteria remained unstained 30 minutes after FM4-64 staining. After staining for more than 2 hours, endogenous bacteria also became stained (data not shown). This result supports previous work showing that FM4-64 staining is time dependent [34]. The short staining was useful to distinguish exogenous and endogenous bacteria. In addition, FM4-64 diffusion was limited because of functional barrier of a plasma membrane, indicating that the colonized epidermal cell was still alive. Three days after inoculation, exogenous bacteria had colonized and adhered to the epidermal cell while endogenous bacteria moved vigorously (Figure 5A, Movie S6). Also, while imaging epidermal cells, we observed bacteria migrating to neighboring cells of the plant host (Figure S1). At 9 days post inoculation, bacterial colonization was observed incide both the apoplast and epidermal cells (Figure 5B). Further cross-sectional observation by the CLMS in the central vein from the surface to the bundle sheath cells (Figure 6, Movie S7) revealed that bacteria also colonized at the bundle sheath cells (Figure 6D) in addition to the epidermis (Figures 6A, B) and apoplast (Figure 6C). Taken together, the *Xcc* entered the xylem vessel, moved quickly in the xylem vessel and multiplied explosively - not only in the xylem vessel but also in the bundle sheath cells, apoplast, and inside the epidermal cells. Other bacteria that did not enter the xylem vessel also moved cell-to-cell and proliferated in the epidermal cells (Movie S2).

### Bacterial Behavior Inside the Xylem Vessel

**Figure 7:**
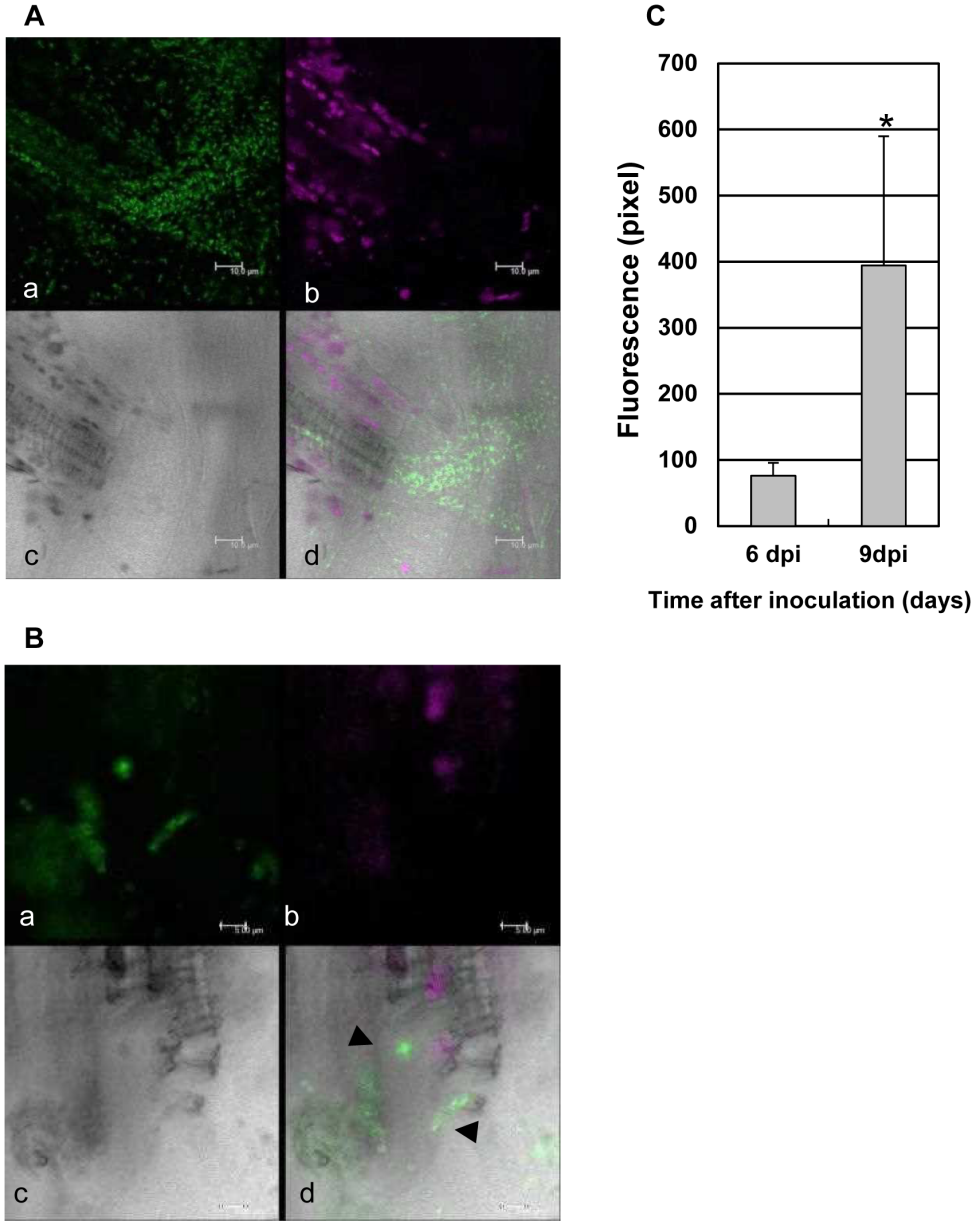
AcGFP-expressing *Xcc*MAFF106712 proliferated and agglomerated inside the xylem vessel of a compatible plant. The leaf surface layer was peeled off and the xylem vessel was extracted; bacteria which escaped the vessel were imaged. (A) Confocal image taken 6 days after inoculation. AcGFP-expressing *Xcc*MAFF106712 proliferated, but no bacterial aggregates formed. Bars, 10 μM. (B) Confocal image taken 9 days after inoculation. Black arrows indicate large bacterial aggregates in a vessel. Bars, 5 μM. (A, B) a, green fluorescence; b, chlorophyll autofluorescence; c, bright field; d, merged image. (C) Size of aggregates at the indicated time was estimated by fluorescence area using ImageJ. More than 30 aggregates from a vessel (n = 8) were measured and the difference between 6 and 9 dpi was significant (*P<0.01, Student’s t test).

To investigate in detail the distribution of bacteria inside the vasculature, the base of the central vein of plants 6 and 9 day post inoculation was cut off and bacteria inside the xylem vessel were discharged to a prepared slide and observed by CLSM (Figure 7). Real-time imaging of showing release of bacteria from the xylem vessel is shown in Movies S8, S9. The bacteria seemed to be aggregated inside the xylem vessel 9 days post inoculation (Figures 7B, S2, Movie S9), with the size of the aggregates depending on each xylem vessel. Notably, the size of the aggregates within a single xylem vessel was relatively consistent. In contrast, bacteria from a xylem vessel only 6 days post inoculation (Figure 7A, Movie S8) seemed not to make such bacterial aggregates, but rather to remain as single cells. The size of bacterial aggregates was measured as pixels of the area showing AcGFP fluorescence, demonstrating the size of the aggregates dramatically increases between 6 and 9 days (Figure 7C). We also noted that the released bacterial aggregates did not move, while single bacteria moved vigorously (Movie S10). At 6 days post inoculation, the infected site in the xylem vessel appeared transversely extended (Movie S4) and most of the bacteria did not move (Movie S3). From these observations, we conclude that active single bacteria with high motility move freely inside the xylem vessel, and upon finding a suitable niche (such as one with a low abundance of other bacteria), they adhere to the plant cells and starts multiplying.

### Real Time Imaging of Type III Mutant Bacteria Inside the Plant

**Figure 8:**
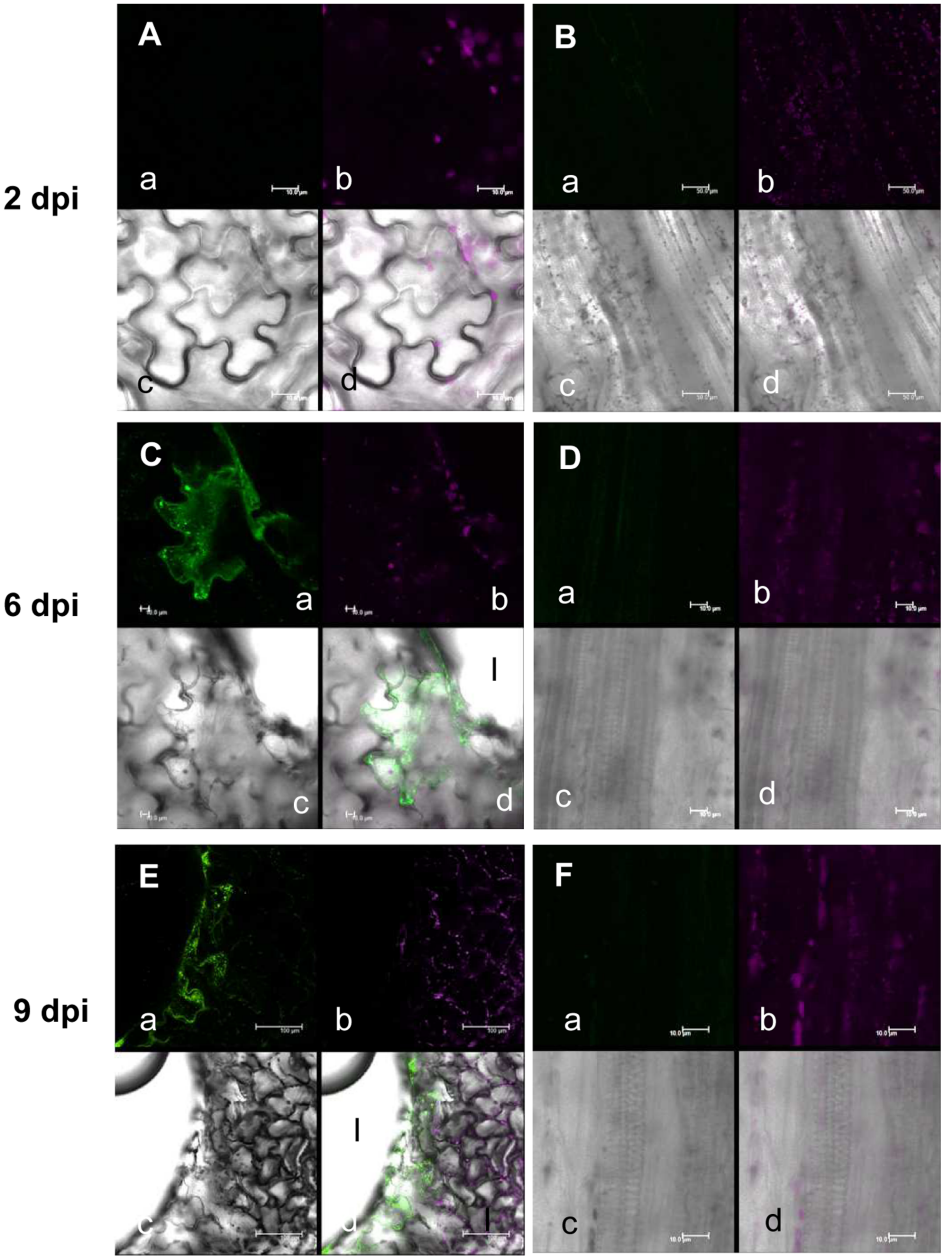
AcGFP-expressing *Xcc*MAFF106712Δ*hrcC* do not colonize a Col-0 plant. Localization of AcGFP-expressing *Xcc*MAFF106712Δ*hrcC* (A to F) in an Arabidopsis Col-0 plant. Leaf surface (A, C and E), central vein 100 μm away from the inoculation site (B) and the xylem vessel of the central vein (D, F) were observed by CLSM. Photographs were taken at 2 days (A, B), 6 days (C, D) and 9 days (E, F) after inoculation. Bacteria only proliferated inside cells adjacent to the inoculation site (I in panel C-d and E-d). Location indicated in Figure 2; i, (A, C and E); ii, (B, D and F). a, green fluorescence; b, chlorophyll autofluorescence; c, bright field; d, merged image. Bars, 10 μM (A, C, D and F); 20 μM (E); 50 μM (B).

We also generated the type III mutant (Δ*hrcC*) of the Col-0 compatible *Xcc* MAFF106712 bacteria harboring the AcGFP plasmid. The inoculation of AcGFP-expressing *Xcc*MAFF106712Δ*hrcC* did not cause any visible symptoms or plant growth defects (data not shown) identical to that of untagged *Xcc*MAFF106712Δ*hrcC* (Figure 2B). As shown in Figure 8, GFP fluorescent were not detected at leaf epidermal cell (Figure 8A) or at surface cells on veins (Figure 8B) around the inoculation site 2 days after inoculation. At 6 days, AcGFP fluorescence was observed only at the cells directly at or adjacent to the inoculation site (Figure 8C), but no GFP fluorescent was detected at the xylem vessel (Figure 8D). Nine days after inoculation, limited fluorescence was detected around the inoculation site (Figure 6E) and GFP fluorescent was never detected in the xylem vessel (Figure 6F). These data indicate that although type III mutant bacteria are able to grow inside a cell, movement and colonization in the ylem vessel is strictly prevented.

### Real Time Imaging of the Incompatible Interaction

**Figure 9:**
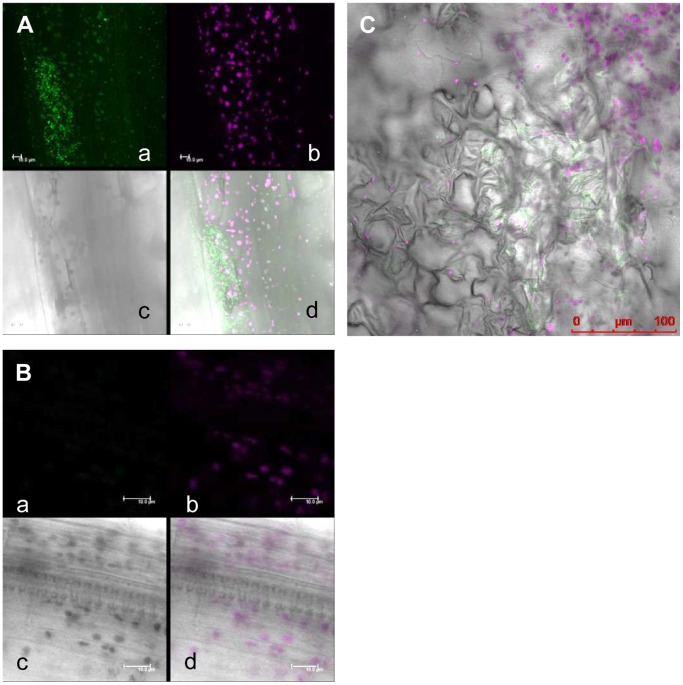
AcGFP-expressing *Xcc*MAFF106712 do not colonize a Sf-2 plant. Localization of AcGFP-expressing *Xcc*MAFF106712 in the incompatible Sf-2 plant was determined by green fluorescence 6 days after inoculation of epidermal cells at the central vein (A), xylem vessel (B) epidermal cells (C). Location indicated in Figure 2; i, (C); ii, (A, B). Bacteria did not proliferate in the xylem vessel (B). Epidermal cell shrinkage was observed where bacteria localized (C). a, green fluorescence; b, chlorophyll autofluorescence; c, bright field; d, merged image. Bars, 5 μM (AB); 100 μM(C).

The *Xcc*MAFF106712 strain is incompatible with the *Arabidopsis* ecotype Sf-2 (Figure 1). AcGFP-expressing *Xcc*MAFF106712 was inoculated on Sf-2 plants and fluorescence was observed by CLSM (Figure 9). We observed bacterial growth inside leaf epidermal cells (Figure 9A), but bacterial growth in the xylem vessel was not detected (Figure 9B). In addition, some of the epidermal cells showed a shrunken phenotype (Figure 9C), suggesting cell death.

### Visualization of Cell Death

**Figure 10:**
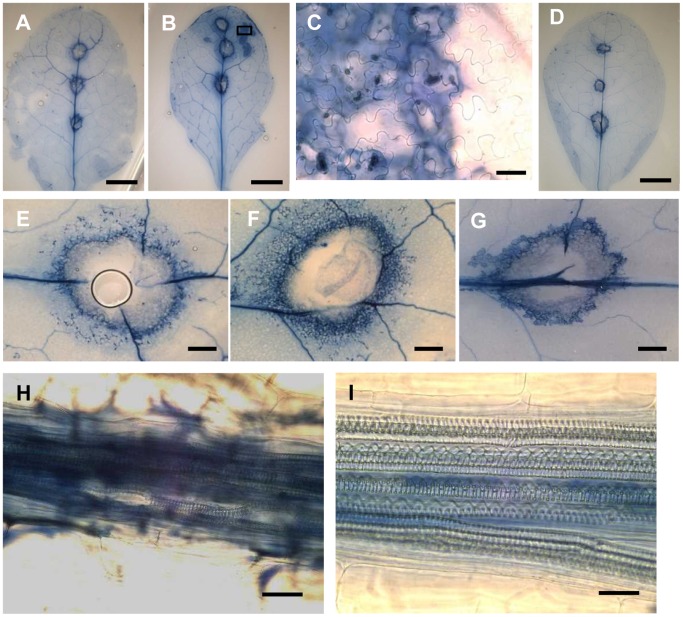
Visualization of cell death. Trypan blue staining of Col-0 (A, E and H) and Sf-2 (B, C, F and I) plants 8 days after AcGFP-tagged *Xcc*MAFF106712 inoculation. *Xcc*MAFF106712Δ*hrcC* in a Col-0 plant 8 days after inoculation (D, G). Shown are: Whole leaf observed by stereomicroscope (A, B and D), an infected area at high magnification (C showing a zoom of the black rectangle in B) by optical microscope, the inoculation wound (E, F and G) by optical microscope, xylem vessel (H, I) by an optical microscope. Bars, 5 mm (A, B and D); 20 μM (C); 1 mm (E, F and G); 10 μM (H, I).

In order to detect cell death, infected leaves were stained with trypan blue and observed by light microscopy (Figure 10). For Col-0 inoculated by *Xcc*MAFF106712, stained cells were observed at the area surrounding the inoculation site (Figures 10A, E) as well as some transversely extended cell. With Sf-2, on the other hand, as shown in Figures 10B, C and F, more distant cells as well as those surrounding the infected site were stained. This suggests that in Sf-2, the HR-like response around the infected site may have prevented further spreading. In the case of type III mutant inoculation of Col-0, stained cells were restricted to the area surrounding the inoculation (Figure 10G). At the base of the central vein, nearby cells of the xylem vessel of Sf-2 (Figure 8G) were not stained, while the same cell type in Col-0 was strongly stained (Figure 8F). This indicates that, in Col-0, the massive proliferation of bacteria in the cells surrounding the xylem vessel (bundle sheath cells) (Figure 8G) leads to cell death.

## Discussion

We selected for study *Xcc* strain MAFF106712, which is able to cause disease on Col-0. The type III repertoire of this strain showed it was missing one of the core type III effectors, XopF1, and two variable type III effectors, XopAC and XopX1 (Figure 1). XopAC has been reported previously as a major avirulence gene of *Xcc* in Col-0 [28], [35]. Supporting this idea, *Xcc*MAFF302026, which is missing most of the type III effectors but retains XopAC, is avirulent on Col-0, yet is virulent on Sf-2 plants (Figure 1). On the other hand, both *Xcc*MAFF106749 and MAFF301964 lack XopAC but still are avirulent in Col-0, which suggests the existence of other factors, in addition to XopAC, that trigger avirulence in Col-0. We have not tested all the type III effectors of *Xcc*, so further research will be needed to determine which effectors cause avirulence. Interestingly, in the case of the bacterial phytopathogen *Pseudomonas syringae*, Sf-2 has been reported to be resistant to a number of *P. syringae* strains while Col-0 is compatible for many of them [36], in contrast to the pattern we observe with *Xcc*. To solve the puzzle of this inverse pathogenicity between *Xanthomonas* and *Pseudomonas* in Col-0, further molecular breeding analysis may help. There is a curious report that a single QTL explained most of the variation (77%) in *P. syringae* susceptibility between accessions Col-0 and Sf-2 [36].

In the compatible interaction (Col-0 and *Xcc*MAFF106749), bacteria spread explosively in the xylem vessel following wounding inoculation (Figures 3, 4). When we inoculated the bacteria without wounding, bacteria growth and disease phenotype shown in Figure 2 did not occur (data not shown). Presumably, *Xcc*MAFF106749 can not enter through the stomata or cuticle. This agrees with the observation that *Xcc* enters the leaf mainly via hydathodes at the leaf margin or wounding [27]. This is contrast to another vasculactural pathogen *Ralstonia solanacearum* that enter Arabidopsis root wituout any openings [17]. Bacteria can enter the xylem vessel easily through the wounding in our experimental condition. Although the motility and growth ability of the type III mutant were not changed *in vitro* (data not shown), the rare colonization in the xylem vessel (Figures 8C, D and F) supported the knowledge of the indispensable role of the type III effectors (T3E) in the plant-microbe interactions [10]–[12].

*Xcc*MAFF106712 can actively move against the normal vascular flow (Figures 3, 7). The water uptake rates of Arabidopsis roots, and water transport within xylem vessels, is estimated to be 478 and 928 μm^3^s^−1^ [37]. Based on our measurements, because the central vein diameter at the base of the leaf is between 5 and 10 μm, the water flow rate should be between 6.3 and 50 μms^−1^. We estimate migration speed of *Xcc* away from the infection site to be between 0 and 25 μms^−1^ (Movie S8), thus bacteria can move against the water flow. It is possible, however, that the flow rate is slower than our estimates due to strategic blockage by the bacterial aggregates. Moreover, since the xylem vessel is made of dead cell, presumably plant basal defense in the xylem vessel is much less efficient in inhibiting bacterial multiplication.

Little is known about the mechanism of a bacterial growth inside the living cells (Figures 4D, G, 5, 6, Movies S1, S2) and bacterial movement to adjacent cells (Figure S1). These results indicate that the pathogen is biotrophic. Other *Xanthomonas* pathogens such as *X. citri* pv. *citri*, responsible for citrus canker, are also characterized as biotrophic pathogens [38]. Similarly, *Xcc* appears to maintain this characteristic. It is well known Gram-negative human pathogens *Shigella* and *Salmonella* multiply intracellularly and spread to neighboring cells [39]. In our experimental condition, *Xcc* showed a similar movement to those human pathogens. Re-organization of actin polymerization stimulated by type III effector and following endocytosis is required for their penetration [39]. It may be possible that *Xcc* has a similar activity towards the plant cell wall - more than 40 cell wall degrading enzymes are encoded by *Xanthomonas* [23], and some of them known to affect the pathogenicity. It is well known that an actin cytoskeleton is required for receptor-mediated endocytosis of ligands including the flagellin receptor FLS2 [40]. Moreover, the actin cytoskeleton in epidermal cells is required the host-cell MAMP receptor kinase complex, including FLS2, BAK1 and BIK1[41]. An interesting question is might *Xcc* enter the epidermal cell via endocytosis? The phenomena we observed in the *Xcc*-Arabisopsis interaction would be a nice source for future study.

Bacteria colonized the bundle sheath cells (Figure 6, Movie S7) which showed cell death (Figure 10H). In addition, infected lesions in the xylem vessel appeared extended transversely (Figure 4E, Movie S4). Those may have been caused by several cell wall degrading enzyme including lipase/esterase (LipA), cellulase, xylanase, and cellobiosidase, which are present throughout *Xanthomonas* species [42]–[44]. These enzymes in *Xcc* might enable the spread of bacteria from one vessel to another. In a study of a vascular pathogen, *R. solanacearum*, the bacteria enter into the vascular cylinder by degrading of the two pericycle cells, and they also moves from vessel to vessel by digesting the pit membrane between adjacent vessels [17]. In the case of *Pseudomonas*, it had been understood that the pathogen multiplied not inside the mesophyll cells but in the intercellular space [13]. However, an important study using *P. syringe* and non-host *N. benthamiana* showed that *P. syringae* moved actively inside the xylem vessel even though its main growth location was apoplast. Moreover, the movement was dependent on syringolin A, a small molecule proteasome inhibitor produced by the pathogen [45]. To our knowledge, no such small compounds secreted by *Xcc* have been shown, though proteomic analysis of the pathogen may be worthwhile. Since XopJ was reported to interacts with a proteasome component to suppress the proteasome interferes with SA-dependent defense response [46], it possibly acts in a similar way as syringolin A in *Xanthomonas*.

Most of the pathogen in the xylem vessel (Movie S3) and apoplast (Figures 5, 6) seemed to adhere to the plant host (Movie S3). It is well studied that the pathogenicity of *Xanthomonas* depends on the adhesive role of the extracellular polysaccharides (EPS) such as xanthan [1], [47]. Diffusible signal factor (DSF) from *X. campestris* is also required for forming aggregates and for full virulence [48]. In the study they showed biofilm formation using GFP tagged Xcc in the chambered slides. Further study confirmed that DFS suppressed stomatal innate immunity [49].

For the incompatible interaction of *Xcc*MAFF106712 with Sf-2, spreading to the cells adjacent to the infected site was strongly inhibited by plant HR-like resistance (Figures 7C, 8B and C), and invasion of the xylem vessel was also inhibited (Figure 7B). As shown in Figure 2A, the number of colony forming units 3 days after inoculation in Sf-2 was comparable to that of Col-0. These results suggested that the pathogen can multiply inside the epidermal cells during first 3 days of inoculation in both incompatible and compatible infections, and the ability of the plant defense system to resist infection becomes most apparent 3 days after inoculation in our experimental conditions. Although we have no genetic evidence for the interaction of an R-gene in Sf-2, or an avirulence protein in *Xcc*MAFF106712, observation of HR-like plant responses at the inoculation site strongly suggests a gene-for-gene defense.

In order to develop an effective pathogen regulatory strategy, it is important to prevent formation of these bacterial aggregates (Figure 7). In addition, counting colony forming units on a petri plate is broadly used as a measurement method of bacterial numbers inside the host cell; we note that efficient agitation is required to break the bacterial aggregates before plating to accurately quantify infection, especially at the late stage of the infection.

As noted above, real time imaging of infected plants by pathogen revealed that the pathogen makes itself at home in the host by attenuating plant defense and changing the structure of host cell through multiple and diverse approaches. Therefore, the development of multiple defense techniques that block these virulence strategies is essential to protect host plants from pathogen invasion.

## Supporting Information

Figure S1 An AcGFP-expressing *Xcc*MAFF106712 bacteria moved to an adjacent cell. The CLSM was programmed to acquire an optical section every 1 s. The original image is shown as Movie S4. White arrow indicates the bacteria moving to the neighboring cell. Shown is the merged image of green fluorescence (green), chlorophyll autofluorescence (red) and bright field. Bar, 10 μm.
(TIF)

Figure S2 AcGFP-expressing *Xcc*MAFF106712 proliferated and agglomerated inside the xylem vessel of a compatible plant. Nine days post inoculation, the leaf surface layer was peeled off and the xylem vessel was extracted; bacteria which escaped the vessel were observed every 3 s using a CLSM. Numbers indicate the elapsed time (s). White arrows indicate large bacterial aggregates in a vessel; black arrows indicate growing aggregates in another vessel. a, green fluorescence; b, chlorophyll autofluorescence; c, bright field; d, merged image. Bars, 5 μM.
(TIF)

Table S1 Oligonucleotide sequences used for PCR-based detection of T3E genes.
(TIF)

Movie S1 Real time confocal image of AcGFP-expressing *Xcc*MAFF106712 on an epidermal cell of Col-0 at 2 days post inoculation.
(AVI)

Movie S2 Real time confocal image of AcGFP-expressing *Xcc*MAFF106712 on a epidermal cell of Col-0 at 3 days post inoculation.
(AVI)

Movie S3 Real time confocal image of AcGFP-expressing *Xcc*MAFF106712 in the xylem vessel of Col-0 at 6 days post inoculation.
(AVI)

Movie S4 Confocal images of AcGFP-expressing *Xcc*MAFF106712 on Col-0 at 6 days post inoculation at the central vein. Z-stack images were taken every 1 μm and piled up with a software equipped with TCS-SP5.
(AVI)

Movie S5 Confocal images of AcGFP-expressing *Xcc*MAFF106712 on Col-0 at 6 days post inoculation. Z-stack images were taken every 1 μm and piled up with a software equipped with TCS-SP5.
(AVI)

Movie S6 Real time confocal image of AcGFP-expressing *Xcc*MAFF106712 of the epidermal cells in Col-0 at 3 days post inoculation with FM4-64 staining.
(AVI)

Movie S7 Confocal images of AcGFP-expressing *Xcc*MAFF106712 on Col-0 at 9 days post inoculation with FM4-64 staining. Z-stack images were taken every 1 μm and piled up with a software equipped with TCS-SP5.
(AVI)

Movie S8 Real time confocal image of AcGFP-expressing *Xcc*MAFF106712 bursting from the xylem vessel of Col-0 at 6 days post inoculation.
(AVI)

Movie S9 Real time confocal image of AcGFP-expressing *Xcc*MAFF106712 bursting from the xylem vessel of Col-0 at 9 days post inoculation.
(AVI)

Movie S10 Real time confocal image of AcGFP-expressing *Xcc*MAFF106712 after having burst from the xylem vessel of Col-0 at 9 days post inoculation.
(AVI)
