# Supplementary figures and images for: Real Time Live Imaging of Phytopathogenic Bacteria Xanthomonas campestris pv. campestris MAFF106712 in ‘Plant Sweet Home’
Source: PLoS One. 2014 Apr 15;9(4):e94386. doi: 10.1371/journal.pone.0094386 (PMC3988059; doi:10.1371/journal.pone.0094386)

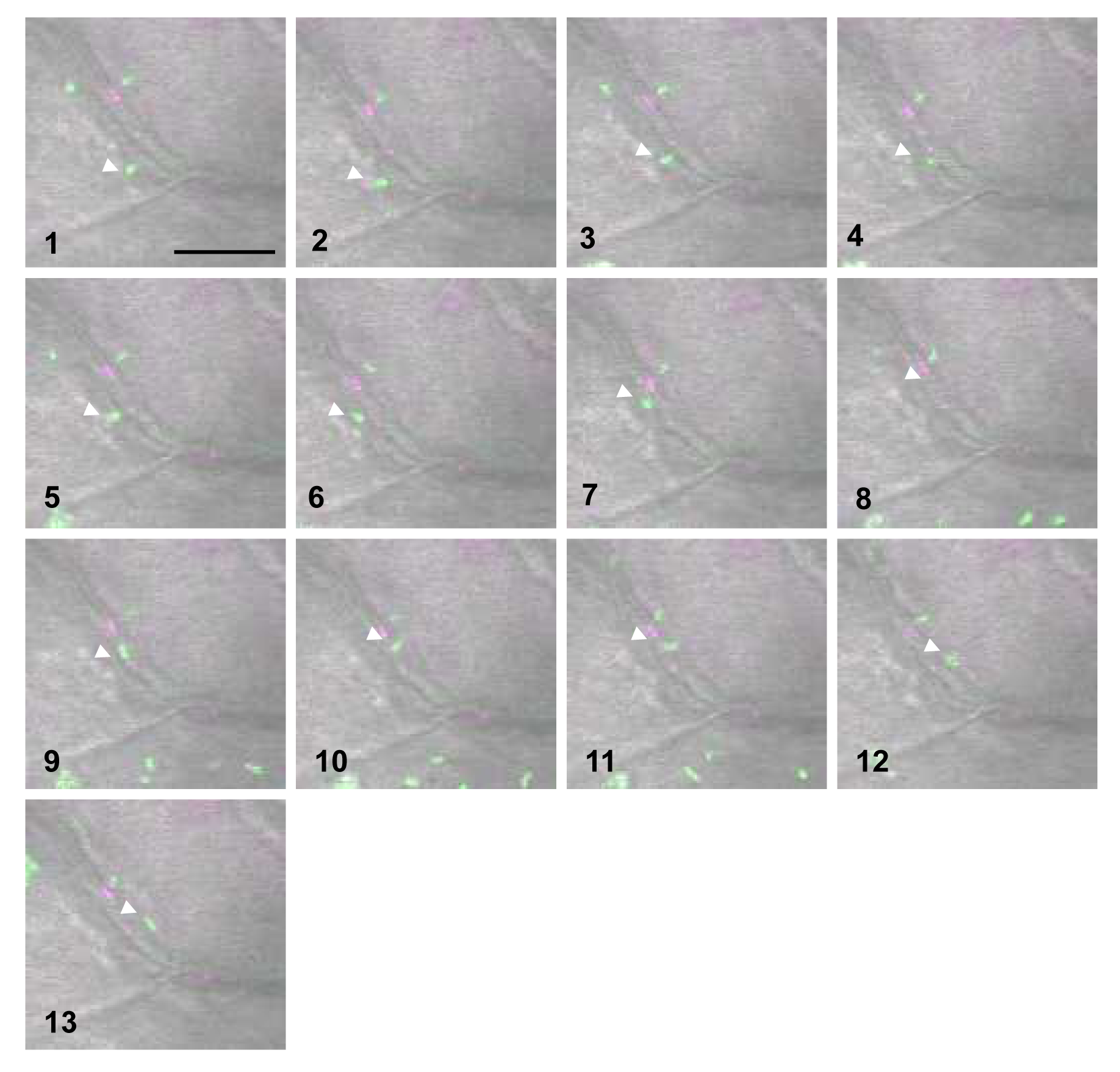

Supplement: Figure S1 — An AcGFP-expressing XccMAFF106712 bacteria moved to an adjacent cell. The CLSM was programmed to acquire an optical section every 1 s. The original image is shown as Movie S4. White arrow indicates the bacteria moving to the neighboring cell. Shown is the merged image of green fluorescence (green), chlorophyll autofluorescence (red) and bright field. Bar, 10 μm. (TIF) [file pone.0094386.s001.tif]

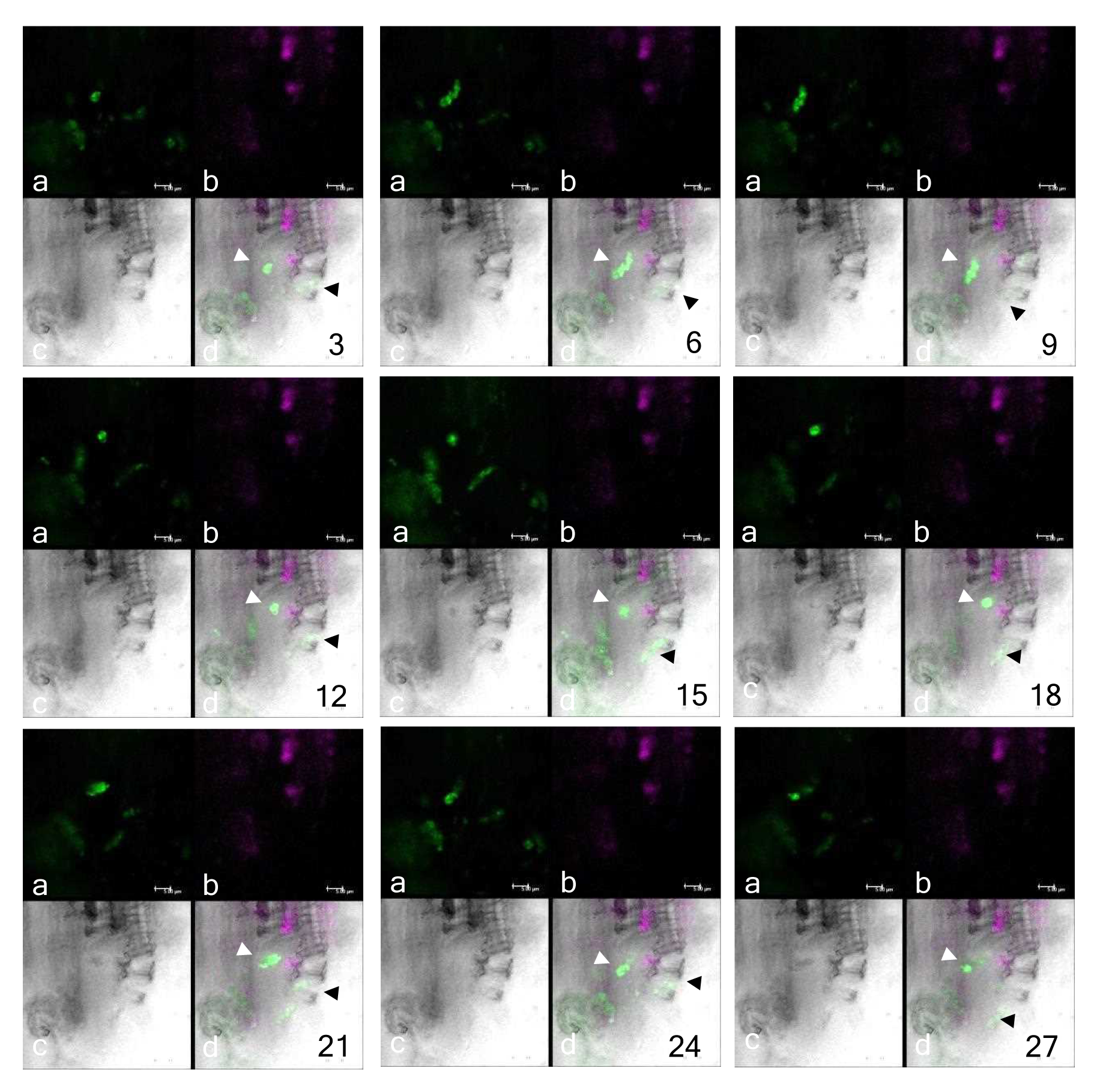

Supplement: Figure S2 — AcGFP-expressing XccMAFF106712 proliferated and agglomerated inside the xylem vessel of a compatible plant. Nine days post inoculation, the leaf surface layer was peeled off and the xylem vessel was extracted; bacteria which escaped the vessel were observed every 3 s using a CLSM. Numbers indicate the elapsed time (s). White arrows indicate large bacterial aggregates in a vessel; black arrows indicate growing aggregates in another vessel. a, green fluorescence; b, chlorophyll autofluorescence; c, bright field; d, merged image. Bars, 5 μM. (TIF) [file pone.0094386.s002.tif]

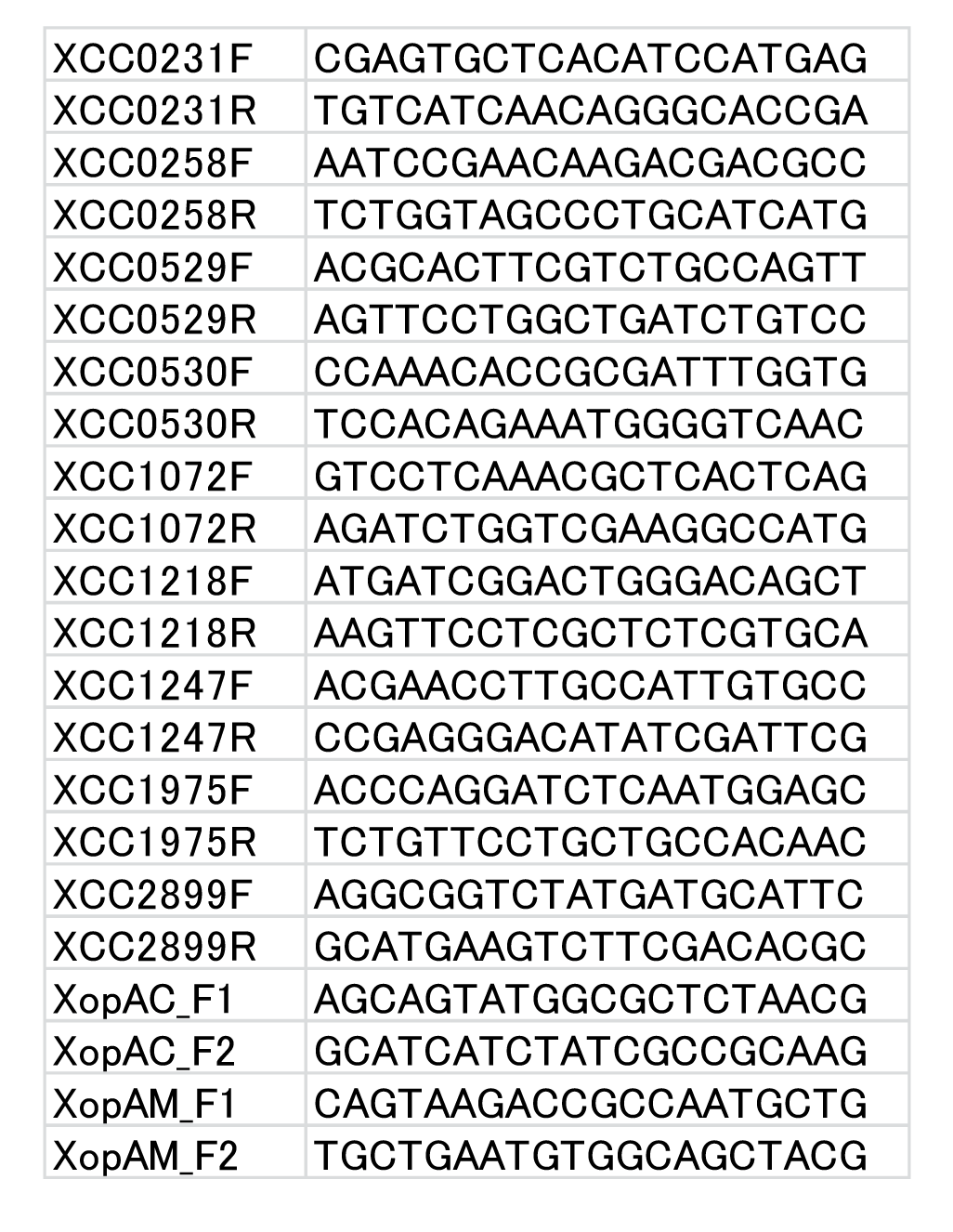

Supplement: Table S1 — Oligonucleotide sequences used for PCR-based detection of T3E genes. (TIF) [file pone.0094386.s003.tif]
